# Cost-Effectiveness of Transcranial Magnetic Stimulation and Virtual Reality-Based Cognitive Remediation for Depressive Symptoms among Cancer Patients: Protocol for a Three-Arm Randomized Controlled Trial

**DOI:** 10.2174/0117450179376750250925051532

**Published:** 2026-03-24

**Authors:** Federica Sancassiani, Martino Belvederi Murri, Clelia Madeddu, Michela Atzeni, Goce Kalcev, Barbara Zaccagnino, Anna Francesca Olivetti, Danila Azzolina, Marco Cruciata, Maria Giulia Nanni, Giulia Cossu, Alessandra Perra, Lorenzo di Natale, Diego Primavera, Massimo Tusconi, Rosangela Caruso, Mauro Giovanni Carta, Luigi Grassi

**Affiliations:** 1 Department of Medical Sciences and Public Health, University of Cagliari, Cagliari, Italy; 2 Center of Liaison Psychiatry and Psychosomatics, University Hospital of Cagliari, Cagliari, Italy; 3 Department of Neuroscience and Rehabilitation, University of Ferrara, Ferrara, Italy; 4 Hospital University Psychiatry Unit, S.Anna Hospital/Local Health Trust, Ferrara, Italy; 5 Oncology Unit, University Hospital of Cagliari, Cagliari, Italy; 6 IDEGO Digital Psychology Society, Rome, Italy

**Keywords:** Depression, Cognitive functions, Quality of life, Cancer, Virtual reality, Repetitive transcranial magnetic stimulation

## Abstract

**Introduction:**

This paper presents a research protocol of a randomized controlled trial aimed to evaluate the feasibility and the cost-effectiveness of non-pharmacological interventions for depressive symptoms, quality of life, depression-related conditions, and cognitive function among patients with cancer. Specifically, repetitive transcranial magnetic stimulation (rTMS) and virtual reality-based cognitive remediation (VR-COG) will be analyzed, alongside standard treatment as usual (TAU), in comparison to TAU alone.

**Methods:**

100 participants will be enrolled: 60 from the Health Trust of Ferrara randomized 1:1:1 to (a) TAU, (b) rTMS + TAU, and (c) VR-COG + TAU, and 40 from the University Hospital of Cagliari randomized 1:1 to (a) TAU and (b) VR-COG + TAU. The inclusion criteria will be as follows: patients aged 18 years and older, both sexes, a diagnosis of oncological disease within the last 5 years in a non-advanced stage, a diagnosis of major depressive disorder according to DSM-5 criteria, and a score of≥14 on the 17-item Hamilton Rating Scale for Depression (HAM-D-17). The VR-COG program will include a series of exercises in virtual sailing scenarios using the software CEREBRUM. The rTMS program will be delivered at 50% of the resting motor threshold. Personalized targets created for each individual will be located at various cortical depths. TAU will include psychiatric visits and psychological counseling. All the interventions will last 3 months, with pre-post evaluation for outcomes of interest and 3-6 months of follow-up.

**Results:**

The results of the trial will be published in international peer-reviewed journals and will be disseminated at international meetings and congresses.

**Discussion:**

The results of this study will be useful for obtaining knowledge for clinical practice regarding the feasibility and cost-effectiveness of innovative therapeutic approaches aimed at treating depression in individuals suffering from oncological pathologies.

**Conclusion:**

The monitoring of the program's cost-effectiveness, encompassing both the screening and intervention phases, will enable policymakers to inform the implementation of this evidence in routine clinical practice.

**Clinical Trial Registration Number:**

The study has been registered on the ClinicalTrials.gov website with ID no. NCT06589544.

## 1 INTRODUCTION

Approximately one in four persons with cancer expresses symptoms of depression [1]. Individuals with cancer and depression are at high risk for severe disability, challenges in returning to work, high risk of suicide, lack of adherence to treatment, poorer prognosis, and decreased survival [2]. Additionally, depression increases the number of visits, hospital admissions, and medical costs [3]. Depression is specifically linked to cognitive deficits in memory, learning, attention, and concentration [4], as well as sleep disturbances, disability, difficulties at work, and suicide risk, all of which worsen the prognosis and increase the risk of relapses or chronicity [2, 5].

Among people with cancer, depression is particularly not recognized or treated. Only 10% of patients receive mental health care. In this sense, primary prevention is practically absent [6] due to the lack of effective screening tools and the poor structure of specialized medical education courses in the Italian and international contexts [7]. Developing personalized screening tools could improve the identification of high-risk patients and their enrolment in specialist treatment pathways at the second or third level [7, 8]. Despite its significant impact on individuals and healthcare systems, substantial gaps remain in the clinical and rehabilitative management of depression among people with cancer. There are no specific guidelines for psychotropic drug usage, and the efficacy of antidepressants is uncertain despite their frequent use [9].

Among non-pharmacological treatments, physical activity is recognized for its preventive and rehabilitative efficacy in addressing mental health complications, particularly concerning depressive and anxiety symptoms [10].

Even if emerging therapeutic strategies, like repetitive transcranial magnetic stimulation (rTMS) and cognitive virtual reality (VR)-based cognitive remediation programs, show promising findings, their cost-effectiveness is understudied [3]. rTMS is already used for the treatment and relapse prevention of depression, both as mono-therapy and as an add-on treatment to antidepressant pharmacotherapy, and it appears effective in improving cognitive performance [11]. However, it has not yet been applied to treat depressive disorders in oncology patients. VR-based cognitive remediation interventions (VR-COG) are designed to improve cognitive functioning, a central feature of depression, even in oncological conditions. VR-COG enhances learning and skill acquisition with better ecological efficiency than traditional cognitive remediation programs [12, 13]. VR approaches are well-received by people with cancer and show promise in reducing anxiety and depressive symptoms [14, 15]. In particular, a VR-COG program has been shown to be feasible and effective not only for improving cognitive functions, but also for reducing depressive and anxiety symptoms, thus enhancing quality of life and improving alexithymia and the regulation of biological and social rhythms in individuals with bipolar disorder [16-22].

This trial aims to evaluate the preliminary effective-ness of highly specialized, nonpharmacological interventions for depressive symptoms among people with cancer and their feasibility according to dropout rates and the proportion of recruited participants among those considered eligible (primary outcomes). The secondary outcomes include the preliminary effectiveness of the interventions in improving quality of life (QoL), addressing depression-related conditions, and enhancing cognitive functions. Specifically, compared to treatment as usual (TAU), rTMS and VR-COG will be tested alongside standard TAU. This trial also aims to evaluate and verify the cost-effectiveness of these treatments (tertiary outcome). This study has been designed as a pragmatic randomized controlled trial, aiming to assess the effectiveness and cost-effectiveness of two non-pharmacological interventions as they would be implemented in real-world oncology settings. We have prioritized external validity and clinical applicability over mechanistic control, using TAU as a comparator to reflect standard care conditions [23].

## 2 MATERIALS AND METHODS

### 2.1 Study Design

The study will involve a randomized controlled trial (RCT) among persons with oncological diseases and mild-moderate depression. The study will include a longi-tudinal, three-arm design with participants assigned by randomization to either an active interventional protocol based on repetitive transcranial magnetic stimulation (rTMS) + treatment as usual (TAU) or virtual reality-based cognitive remediation (VR-COG) + TAU, and to a control setting, treatment as usual (TAU). The main outcomes are expected to show the superiority of each experimental intervention (rTMS + TAU or VR-COG +TAU) over the control condition (TAU). Participants will be randomized, independently, to parallel intervention groups in each of the two units [unit 1, Health Trust of Ferrara (UniFE), with 1:1:1 randomization to (a) TAU, (b) rTMS + TAU, and (c) VR-COG + TAU; unit 2, University Hospital of Cagliari (UniCA), with 1:1 randomization to (a) TAU and (b) VR-COG + TAU] with the stratification for the unit of the recruitment.

The timeline of the different study phases and the protocol flow diagram are presented in Figs. (**1** and **2**), respectively.

### 2.2 Participants and Recruitment

The target population for this study will be subjects with oncological diseases and moderate depression. Inclusion criteria will be as follows: patients aged 18 years and older of both genders; diagnosis of oncological disease in the last 5 years; depression without psychotic symptoms according to Hamilton Rating Scale for Depression (HAM-D-17) score ≥14; and oncological disease in a non-advanced stage (Karnofsky performance status > 80).

Exclusion criteria will comprise current or prior hospitalization in the next 6 months, planned surgery in the next 6 months, suicidal ideation, substance use, history of significant head trauma, neurological disorders, intellectual deficits, recurrent seizures resulting from head trauma or conditions lowering seizure threshold, concurrent use of medications that increase the risk of epileptic seizures (*e.g.*, antipsychotics, tricyclics, theophylline), glaucoma, retinal detachment, or other serious vision impairments that may prevent the use of virtual reality technology and severe problems with autonomous ambulation.

**Fig. (1):**
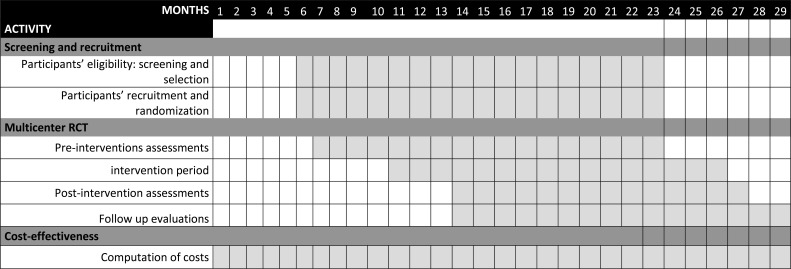
A Gantt chart showing the timeline of the activities.

**Fig. (2):**
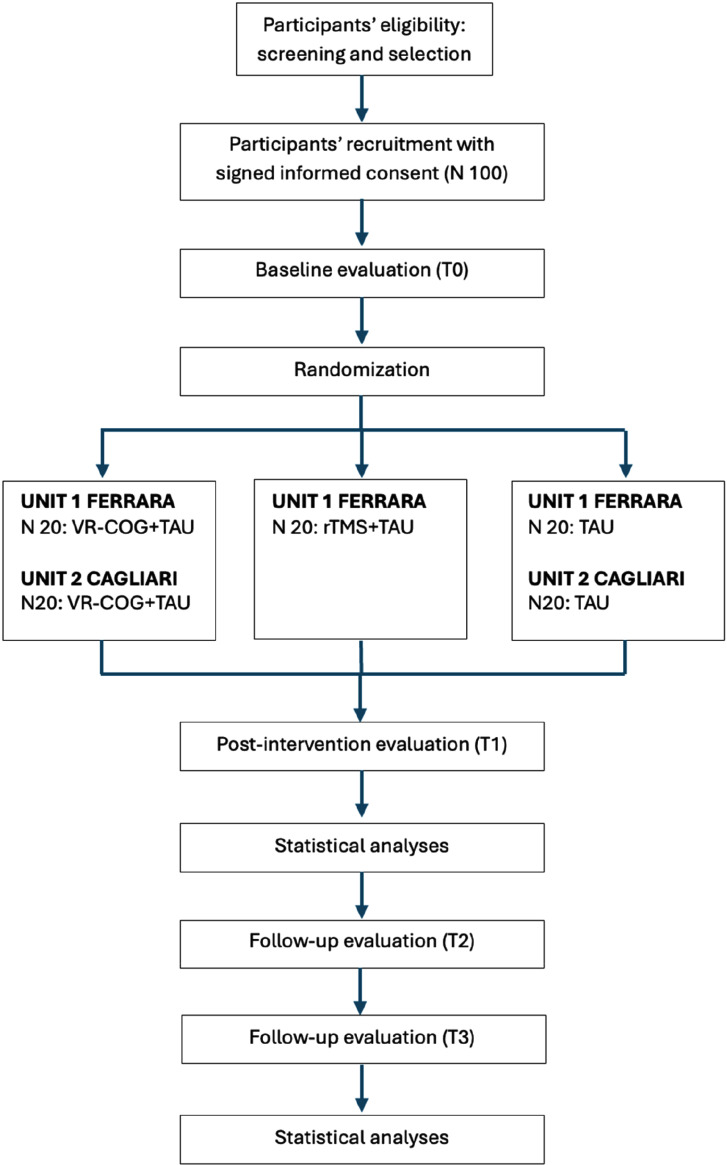
Flow chart of the RCT.

According to the inclusion/exclusion criteria, recruitment will continue until the target sample size is reached, allowing for randomization and the conduct of interventions (Figs. **1** and **2**).

### 2.3 Outcomes

The following outcome measures will be established for each participant based on clinical relevance, Cochrane Collaboration indications, and cost-effectiveness studies.

The primary outcomes will include depressive symptoms and the study’s feasibility indicators (acceptability, tolerability, side effects, or secondary effects due to the use of technological devices).

The secondary outcomes will comprise quality of life, cognitive functions (immediate memory, working memory, phonemic verbal fluency, delayed memory, psychomotor speed, executive function, selective attention), and other depression-related conditions (demoralization, embitter-ment reactions to negative life events, psychological distress, insomnia, social and biological rhythms dysregulation, disability level, and alexithymia).

The tertiary outcome will include the cost-effectiveness of the nonpharmacological interventions (VR-COG, rTMS) in addition to TAU compared to TAU alone.

### 2.4 Evaluation Tools and Evaluation Timeline

To assess the primary outcomes, the tools and evaluation procedures that will be used are as follows:

- Hamilton Depression Rating Scale, comprising 17 items (HAM-D-17) [24], is a clinician-administered tool widely used to assess the severity of depressive symptoms, which evaluates mood, guilt, suicide ideation, insomnia, and somatic symptoms over the preceding week. Each item is rated on a 3- or 5-point scale, with higher scores indicating greater depression severity. Its reliability and validity have been extensively established. The scale will be administered according to the following timeline: T0 (0 months-baseline), T1 (3 months-post-intervention), T2 (3 months after T1), and T3 (6 months after T1).
- Drop-out rates, the proportion of recruited participants among those considered eligible, and secondary or side effects due to virtual reality or repetitive transcranial stimulation will be the measures employed to assess the feasibility of the study. These will be estimated according to the following timeline: T0 (0 months-baseline), T1 (3 months-post-intervention), T2 (3 months after T1), and T3 (6 months after T1). Side and secondary effects will be assessed through the Simulator Sickness Questionnaire [25], a self-report questionnaire including 16 items that evaluate the frequency of unwanted effects due to virtual reality technologies, such as nausea, dizziness, headaches, eye strain, *etc.* The TMSens_Q questionnaire [26] will be used to evaluate various unintended sensations and adverse effects, ranging from mild side effects to serious adverse events related to the use of rTMS. The questionnaire will be administered according to the following timeline: T0 (0 months-baseline) and T1 (3 months-post-intervention). Figure **1** provides a summary of the timeline.

To assess the secondary outcomes, the following tools and evaluation procedures will be employed:

- EuroQol (EQ)-5D is a self-report questionnaire to assess health-related quality of life [27]. It encompasses two parts: a descriptive one and a visual analogue scale (VAS). The descriptive part includes five dimensions, including mobility, self-care, usual activities, pain/ discomfort, and anxiety/depression, with each rated on a three-level (EQ-5D-3L) or five-level (EQ-5D-5L) scale, indicating no problems to extreme problems. The VAS records the respondent's self-rated health on a scale from 0 (worst health imaginable) to 100 (best health imaginable). It demonstrates good internal consistency and reliability, with Cronbach's alpha values ranging from 0.6 to 0.85, varying based on the population and clinical context.
- The SF-12 Health Survey [28] is a brief version of the widely used SF-36, designed to measure health-related quality of life. It consists of 12 items that cover physical and mental health domains. The SF-12 assesses the impact of health on daily functioning, pain, energy, emotional well-being, and role limitations due to physical or emotional problems. It demonstrates good internal consistency and reliability, with Cronbach's alpha values typically exceeding 0.80 for both physical and mental health domains.

Both of the above questionnaires will be administered according to the following timeline: T0 (0 months-baseline), T1 (3 months-post-intervention), T2 (3 months after T1), and T3 (6 months after T1).

- Screen for Cognitive Impairment in Psychiatry (SCIP) [29] is a brief and validated neuropsychological tool designed to assess cognitive functioning in individuals with psychiatric disorders. It evaluates key cognitive domains, including verbal learning, working memory, processing speed, and executive functions, making it particularly useful in detecting cognitive impairments associated with conditions, such as schizophrenia, bipolar disorder, and major depressive disorder. The SCIP is time-efficient, typically requiring 10-15 minutes to administer, and has demonstrated high reliability, with Cronbach's alpha values ranging from 0.74 to 0.90, depending on the population and cognitive domain assessed. The scale will be administered according to the following timeline: T0 (0 months-baseline), T1 (3 months-post-intervention), T2 (3 months after T1), and T3 (6 months after T1).
- Trail Making Test [30] is a widely used neuropsychological assessment tool designed to evaluate cognitive flexibility, visual attention, and processing speed. It consists of two parts as follows: TMT-A, which requires participants to connect numbered circles sequentially, and TMT-B, which alternates between numbers and letters, assessing more complex executive functions, such as task-switching and set-shifting. Reliability studies have reported Cronbach's alpha values ranging from 0.70 to 0.89, depending on the sample and testing conditions. The questionnaire will be administered according to the following timeline: T0 (0 months-baseline), T1 (3 months-post-intervention), T2 (3 months after T1), and T3 (6 months after T1).
- Digit Span [31] is a neuropsychological test that evaluates working memory, attention, and immediate verbal memory. It consists of two components as follows: digit span forward, which evaluates simple attention and memory span by requiring participants to repeat a sequence of numbers in the same order, and digit span backward, which assesses working memory and cognitive flexibility by requiring participants to repeat the sequence in reverse order. Reliability studies have reported Cronbach's alpha values ranging from 0.70 to 0.90, indicating good internal consistency across different populations and testing contexts. The test will be administered according to the following timeline: T0 (0 months-baseline), T1 (3 months-post-intervention), T2 (3 months after T1), and T3 (6 months after T1).
- Stroop Test [32] is a widely used neuropsychological test that measures cognitive control, selective attention, and processing speed. It evaluates the ability to inhibit automatic responses and resolve cognitive interference by requiring participants to identify the ink color of a word that may denote a different color name (*e.g.*, the word “red” printed in blue ink). The test typically includes three conditions: “reading color names”, “naming colored blocks”, and “naming the ink color of incongruent color-word pairs”. The Stroop effect, reflected in the increased time or errors during the incongruent condition, provides insights into executive functioning and attentional control. Reliability studies have reported Cronbach's alpha values ranging from 0.72 to 0.91, depending on the test version and population studied. The test will be administered according to the following timeline: T0 (0 months-baseline), T1 (3 months-post-intervention), T2 (3 months after T1), and T3 (6 months after T1).
- Frontal Assessment Battery (FAB) [33] is a brief neuropsychological tool designed to evaluate executive functioning and frontal lobe cognitive abilities. It consists of six subtests assessing different aspects of executive function: conceptualization, mental flexibility, motor programming, sensitivity to interference, inhibitory control, and environmental autonomy. Studies have reported Cronbach's alpha values ranging from 0.78 to 0.90, indicating good internal consistency across various populations. The test will be administered according to the following timeline: T0 (0 months-baseline), T1 (3 months-post-intervention), T2 (3 months after T1), and T3 (6 months after T1).
- Rey's Word Test [34] is a neuropsychological assessment tool used to evaluate verbal memory and malingering by assessing an individual’s ability to recall and recognize words presented in a list format. The Cronbach’s alpha falls within a range of 0.70 to 0.85, depending on the specific population and administration conditions, indicating acceptable to good internal consistency. The test will be administered according to the following timeline: T0 (0 months-baseline), T1 (3 months-post-intervention), T2 (3 months after T1), and T3 (6 months after T1).
- Matrix test [35] is a cognitive assessment tool designed to evaluate non-verbal reasoning, abstract thinking, and problem-solving abilities. It typically involves identifying patterns or completing sequences in visual matrices. The reliability of the Matrix test, as indicated by Cronbach’s alpha, generally ranges from 0.80 to 0.90, reflecting good to excellent internal consistency. The test will be administered according to the following timeline: T0 (0 months-baseline), T1 (3 months-post-intervention), T2 (3 months after T1), and T3 (6 months after T1).
- Demoralization Scale (DS) [36] is a 24-item self-administered questionnaire with four subscales: discouragement, loss of meaning/purpose, dysphoria, and sense of failure. It measures feelings of helplessness, loss of meaning, and subjective incompetence, often associated with psychological distress in medical and psychiatric contexts. The questionnaire demonstrates high reliability, with Cronbach’s alpha typically ranging from 0.85 to 0.95, indicating excellent internal consistency across diverse populations. It will be administered according to the following timeline: T0 (0 months-baseline), T1 (3 months-post-intervention), T2 (3 months after T1), and T3 (6 months after T1).
- Post-traumatic Embitterment Disorder Self-rating Scale (PTED Scale) [37] is a 21-item tool designed to assess the symptoms of post-traumatic embitterment disorder (PTED), a condition that emerges following a significant, often unfair, negative life event. This disorder is characterized by persistent feelings of embitterment, resentment, and anger, which may lead to emotional and social difficulties. The Cronbach’s alpha coefficient indicates good internal consistency, ranging from 0.85 to 0.95. The scale will be administered according to the following timeline: T0 (0 months-baseline), T1 (3 months-post-intervention), T2 (3 months after T1), and T3 (6 months after T1).
- Brief Symptom Inventory (BSI) [38] is a widely used self-questionnaire designed to evaluate a range of psychological symptoms and distress. It consists of 53 items, which assess nine primary symptom dimensions, including anxiety, depression, and somatization, as well as a global severity index (GSI) that measures overall psychological distress. The internal consistency of the BSI is high, with Cronbach’s alpha values ranging from 0.70 to 0.90 across various subscales, indicating strong reliability in measuring psychological symptoms. The questionnaire will be administered according to the following timeline: T0 (0 months-baseline), T1 (3 months-post-intervention), T2 (3 months after T1), and T3 (6 months after T1).
- Insomnia Severity Index (ISI) [39] is a self-report questionnaire to assess the severity of insomnia symptoms. It consists of 7 items that evaluate the degree of difficulty with sleep initiation, sleep maintenance, early morning awakening, and the impact of these symptoms on daily functioning. The scale has demonstrated strong internal consistency, with Cronbach’s alpha values ranging from 0.74 to 0.90. The questionnaire will be administered according to the following timeline: T0 (0 months-baseline), T1 (3 months-post-intervention), T2 (3 months after T1), and T3 (6 months after T1).
- Biological Rhythms Interview for Assessment in Neuropsychiatry (BRIAN) [40] is a scale consisting of 18 items to assess four areas of circadian rhythm difficulties: sleep, activity, social rhythms, and eating patterns. The scale demonstrates good internal consistency, with Cronbach’s alpha value ranging from 0.80 to 0.90, indicating its reliability as an assessment tool for biological rhythm dysregulation. The questionnaire will be administered according to the following timeline: T0 (0 months-baseline), T1 (3 months-post-intervention), T2 (3 months after T1), and T3 (6 months after T1).
- Activities of Daily Living (ADL) [41] is a questionnaire that evaluates the ability to perform basic self-care tasks and daily activities to estimate the functional status and disability. Its internal consistency is strong, with Cronbach’s alpha value ranging from 0.70 to 0.90. The questionnaire will be administered according to the following timeline: T0 (0 months-baseline), T1 (3 months-post-intervention), T2 (3 months after T1), and T3 (6 months after T1).
- Psychosocial Adjustment to Illness (PAIS) [42] is a self-report instrument to evaluate the psychosocial impact of illness on an individual’s life. It examines seven domains, including social relationships, emotional well-being, work and leisure activities, and family dynamics, in response to chronic illness or medical conditions. It has demonstrated good internal consistency, with Cronbach’s alpha value ranging from 0.80 to 0.95, reflecting its reliability in capturing the psychological and social adjustments with illness. The questionnaire will be administered according to the following timeline: T0 (0 months-baseline), T1 (3 months-post-intervention), T2 (3 months after T1), and T3 (6 months after T1).

Toronto Alexithymia Scale 20-item (TAS-20) [43] is a self-report questionnaire designed to assess the degree of alexithymia. It consists of 20 items that measure three key dimensions of alexithymia: difficulty identifying feelings, difficulty describing feelings, and externally oriented thinking. The instrument demonstrates strong internal consistency, with Cronbach’s alpha ranging from 0.80 to 0.90. The questionnaire will be administered according to the following timeline: T0 (0 months-baseline), T1 (3 months-post-intervention), T2 (3 months after T1), and T3 (6 months after T1). A summary of the timeline is provided in Fig. (**1**).

The tertiary outcome will be measured as cost-effectiveness [3] of the nonpharmacological treatments (VR-COG and rTMS), and it will be computed as the ratio of total costs divided by the mean improvements of quality of life across interventions (TAU, TAU+rTMS, TAU+VRCOG). Total costs will include the costs of the healthcare consultations (visits in the TAU condition), psychotropic drugs, equipment, and staff. Total costs will be computed as the sum of the costs of each intervention, divided by the number of patients. Quality of life improvements will be computed as the difference between the baseline (T0) and the endpoint (T3) quality of life. A summary of the timeline is provided in Fig. (**1**).

### 2.5 Setting

All the activities will be carried out in two facilities of the Italian National Health Service:

1. Interhospital Psycho-Oncology Program, Psychiatry Unit, Mental Health Department for Pathological Addictions, Health Trust Unit of Ferrara, Italy (unit 1).
2. Center of Liaison Psychiatry and Psychosomatic Medicine and Medical Oncology Unit, University Hospital of Cagliari, Italy (unit 2).

### 2.6 Randomization

As shown in Fig. (**2**), the randomization will be conducted at units 1 and 2 after baseline assessment. Randomization will be computer-based and concealed. The people who will conduct the randomization procedure will be blind to the participants’ identities and status and will not receive any information on the participants. Randomization will be performed by blocks of randomized permutations to experimental and control treatments at a 1:1:1 (unit 1) and 1:1 rate (unit 2). Codes will be masked.

### 2.7 Allocation Concealment

At least three different professionals will be present in the two study units: coordinator, evaluator, and clinician. In addition, the study will require the presence of a single person (statistician) for the two units, who will be responsible for the randomization procedures. Recruitment will be done by the study clinician or the clinical facility members, where the screening procedures will be carried out (medical oncologist, psychologist, or psychiatrist). They will propose participation in the trial to interested patients and will obtain their informed consent. Then, the clinicians will perform an initial screening of the inclusion/exclusion criteria and will get in touch with the coordinator if the eligibility requirements are satisfied. They will then reach out to the study's evaluator, who will then perform the baseline assessment. The statistician will perform the randomization procedure after receiving the data required from the coordinator. To ensure individual blinding of the clinical assessments by the evaluator, the randomization list will be shared with the coordinator and the clinician but not with the evaluator.

### 2.8 Blinding

The nature of the interventional procedures anticipated for this study does not permit the activation of a blindness mechanism in the subjects involved. The study will be carried out as a single-blind study. In particular, participants will be contacted by telephone by the evaluator, independently of the coordinator (and possibly the clinician). Participants will be asked not to disclose to the evaluator which therapeutic interventions they are receiving.

### 2.9 Interventions

#### 2.9.1 Control: Treatment as Usual (TAU)

Treatment as usual (TAU) path, following the guidelines of the Italian Association of Medical Oncology (AIOM-SIPO: https://www.aiom.it), is an active comparator and includes an initial psychiatric visit aimed at assessing the presence of psychopathological conditions that may necessitate pharmacological therapy and subsequent psychiatric follow-up. The treatment will also involve psychological counselling (bi-monthly sessions) for 12 consecutive weeks. The duration of each session will be 50 minutes.

#### 2.9.2 Experimental 1: Virtual Reality-based Cognitive Remediation (VR-COG) + Treatment as Usual (TAU)

 The "VR-COG" experimental protocol integrates sailing virtual environments within the CEREBRUM software platform (Idego-Promind, srl, Rome, Italy). It is based on the virtual reality software CEREBRUM (Idego-Promind, srl, Rome, Italy). The virtual scenarios dedicated to the sport of sailing offer four cognitive training exercises, each with three progressively challenging levels of difficulty, designed to train different cognitive functions (*i.e.*, executive functions, motor ability, language). The different degrees of difficulty are designed to adapt to the user's functional diagnosis. Each session, after an initial part of welcome, psychoeducation, and orientation to the instrument, involves alternating virtual reality exercises, positive and corrective feedback, and suggestions of practical homework that the individual should try to do during the day [16-22]. The frequency is 24 sessions, 2 times a week, for 12 consecutive weeks. The duration of each session is 50 minutes.

#### 2.9.3 Experimental 2: Repetitive Transcranial Magnetic Stimulation (rTMS) + Treatment as Usual (TAU)

The rTMS group will receive active rTMS using the MagVenture MagPro X100 System (MagVenture A/S, Denmark) equipped with a MagVenture Cool-B65 A/P double-faced coil and a neuronavigation system (Localite GmbH, Sankt Augustin, Germany). Active rTMS stimulation will be delivered at 50% of the resting motor threshold (rMT). Personalized targets created for each individual will be located at various cortical depths. For safety reasons, the stimulation intensity will never exceed 120% of the rTMS [44, 45]. The frequency will be 24 sessions, 2 times a week, for 12 consecutive weeks of rTMS with the intermittent theta burst stimulation (iTBS) protocol on the left dorsolateral prefrontal cortex (DLPFC). The daily sessions will consist of 3 consecutive sessions of 600 pulses, with each session comprising triplets of pulses at a frequency of 50 Hz, repeated every 200 ms (5 Hz bursts).

#### 2.9.4 Data Management and Security

The main goal of the data management process is to guarantee the timely delivery of high-quality data in order to meet the requirements of appropriate statistical analysis and good clinical practice (GCP) standards [46]. It includes all phases of data management, such as data collection, processing, and application. All clinical data will be collected in a comprehensive longitudinal database to facilitate research procedures. Patient information will be recorded *via* IT (information technology) support (REDCap 8.10.18) with reserved and controlled access [47]. Each participant will be assigned a unique identification number at the start of the study. As well, EDC (electronic data capture) will be developed. This record will be used to design, build, and implement a database for data collection, manage data quality, create automated queries for study monitoring, harmonize data provided by different sources, and export the collected data in formats useful for statistical processing. The study will be conducted following the Declaration of Helsinki [48] and the requirements of all applicable local and international standards, according to data protection laws. Written informed consent from all patients will be obtained before data collection.

#### 2.9.5 Methods to Handle Missing Data

Special efforts will be made to minimize missing data rates and collect data related to the outcomes for participants withdrawing early from the study. This will include proposing evaluations *via* telemedicine platforms or non-video-recorded video calls, or setting up new check-ups at 3, 6, and 9 months.

#### 2.9.6 Adverse Event Reporting

The clinicians in charge of TAU at each appointment will collect, report, evaluate, and manage adverse events or other unexpected effects of the interventions being studied or related to the trial's implementation. Each adverse event will be reported, including details about the type of adverse event, its beginning and end, severity, whether or not it could have been expected, its outcome, and whether or not it was related to the study.

#### 2.9.7 Oversight and Monitoring

The dissemination committee will be formed with representatives from both units. The committee will be chaired by the principal investigator from unit 1, who will be responsible for the final decisions. An interim analysis is planned at 70% of the recruitment. Monthly intra- and inter-centre meetings will be scheduled to monitor the progress of recruitment, the safety of interventions, and data collection. The meetings will take place independently of the sponsor.

### 2.10 Ethical Issue

According to the Declaration of Helsinki's guidelines [48], each study participant will provide their signed informed consent. The processing of personal data used in the healthcare sector will comply with the legislation under Regulation (EU) 2016/679 (General Data Protection Regulation - GDPR). The clinicians responsible for the TAU at units 1 and 2 will collect written informed consent.

The study protocol has been approved by the ethics committee “Area Vasta Emilia Centro della Regione Emilia-Romagna (CE-AVEC)”, Italy (199/2023/Disp/ AUSLFe, 04/04/2023), and by the ethics committee “Azienda Ospedaliero-Universitaria di Cagliari”, Italy (Prot. NP/2023/1566, 06/04/2023).

### 2.11 Statistical Analysis

The baseline data will be summarised as percentage (absolute numbers) for qualitative variables and median (1^st^ and 3^rd^ quartiles) for continuous variables. The group sequential Bayesian design [49] will be used to analyze the study's primary endpoints. Additionally, the first interim analysis will be carried out when 70% of enrolment is reached. The pattern chosen for the evaluation of the risk ratios of the primary endpoint will be defined by a beta-binomial model [50]. Apart from that, a SHELF (Sheffield Elicitation Framework) elicitation paradigm [51] will be used to define the prior probability of remission in the three intervention groups. The elicitation procedure will be carried out using R Shelf software [52]. Non-informative priors will still be used for sensitivity analyses. Upon reaching the interim, the probability that the posterior RR (relative risk) estimates, for the TAU *versus* rTMS or TAU *versus* VR-COG comparison, imply a value greater than 1 and surpass an efficacy boundary established in the interim, will be calculated. If at least one posterior probability for comparison using informative and non-informative priors exceeds the interim boundaries, then the trial will end due to efficacy; otherwise, the trial will continue until the end of the study. If the trial advances, the posterior probabilities for the RRs, with 95% confidence intervals, will be calculated. The intervention will be considered effective if the posterior probability that the RR exceeds 1 is higher than the ultimate effectiveness boundary. Secondary analyses will be performed using generalized Bayesian models by taking into account the interventions as a covariate and reporting 95% posterior confidence intervals. The convergence of the models will be evaluated using trace plots and Gelman statistics. The data will be analysed with an intention-to-treat approach and imputation of missing data according to a Last Observation Carried Forward (LOCF) protocol [53].

#### 2.11.1 Sample Size

Sample size and statistical power will be estimated using a Bayesian group sequential design, incorporating an interim assessment at 70% of enrolment [54]. The primary endpoint of the study will be defined as the comparison of remission rates between the TAU and rTMS groups and the TAU *vs*. VR-COG groups.

**Fig. (3):**
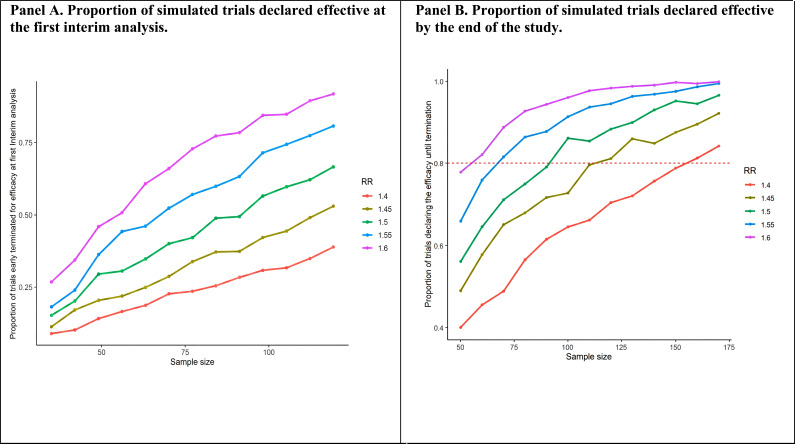
Study design properties.

Literature convincingly supports the efficacy of TMS in major depressive disorder, although not specific to the oncological setting. A recent meta-analysis reported a significant effect on the remission endpoint of rTMS, with an odds ratio of 2.56 (95% CI: 1.73-3.78) [11], corresponding to a risk ratio of 1.5 (95% CI: 1.4-1.6). While no estimate is available on the efficacy of VR-based interventions comparable to VR-COG. Virtual reality-based interventions that do not include cognitive rehabilitation, within oncological settings, have shown effects with a standardized mean difference (SMD) in depressive symptom reduction of -1.11 (95% CI: -3.17 to 0.96) [15]. Considering that traditional (non-VR-based) cognitive rehabilitation for patients with depression, outside of oncology, is associated with a more modest reduction in depressive symptoms (SMD = 0.43; 95% CI: 0.17 to 0.70) [12], a conservative estimate of the effect size would be appropriate. Therefore, an SMD of 0.90, also corresponding to a risk ratio (RR) of 1.5, will be adopted. This design will be explored through a Monte Carlo simulation experiment with 10,000 runs. Each simulation run will assume a scenario with a different combination of effect size and sample size. For each scenario, the proportion of simulated trials correctly identifying at least one effective intervention will be computed, thereby estimating the empirical power of the study in a Bayesian context (Fig. **3**). Specifically, in each run, trial data will be generated under various scenarios assuming a remission rate of 0.6 for the TAU group and a risk ratio (RR) for comparisons with the experimental groups ranging between 1.4 and 1.6 (Fig. **3**). In the simulations, the total sample size n will vary from 50 to 170 patients, with increments of 10 subjects per simulation. The TAU and VR-COG groups will be assigned a sample size twice that of the rTMS group. For example, in a study with 50 participants, 10 patients would be assigned to the rTMS group (available only in Cagliari), 20 to the VR-COG group (Cagliari and Ferrara), and 20 to the TAU group (Cagliari and Ferrara).

For each Monte Carlo iteration, simulated trial data will be generated assuming a binomial distribution for the TAU group, xTAU ∼ Bin(n1 = 1/5 * n,p1 = 0.6), while for the other groups, the distribution will be xVR-COG ∼ Bin(n = 2/5 * n,p2 = 0.6 * RR). Within each iteration, the posterior probability for the RR will be computed using a beta-binomial distribution with a non-informative prior Beta (1,1). This approach has been chosen to keep the study design conservative during the planning phase. The posterior probability estimates for the RR will be obtained by taking the ratio of the posterior estimates of the remission rates between the two intervention arms (*e.g.*, VR-COG *vs*. TAU and rTMS *vs*. TAU).

Using these posterior estimates of the RR, it will be possible to derive the probability that the RR is greater than 1, applying the efficacy boundary method. This approach would allow for the definition of criteria to declare the intervention effective either at the end of the trial or earlier, at the time of interim analyses. The boundaries will be derived from the cumulative distribution function of a normal distribution, based on the frequentist O’Brien and Fleming boundaries, and translated into a Bayesian framework, as suggested in the literature [55]. The efficacy thresholds will be as follows: φ interim/(z=2.8)=0.997 e φ overall/(z=1.98)=0.976.

An interim analysis is planned at 70% of the total enrolment. If the posterior probability of the intervention’s efficacy exceeds the predefined efficacy boundary at the interim analysis, early termination of that intervention arm for efficacy will be considered. A total sample size of 100 participants (70 at interim analysis, allocated as 14 to rTMS, 28 to TAU, and 28 to VR-COG) would allow for a 41% probability of declaring the intervention effective at interim, assuming a risk ratio of at least 1.5. The evaluation of the other intervention will continue until the completion of the planned enrollment.

In conclusion, based on the simulation study, a total sample size of 100 participants would allow for a true positive rate of 81%, *i.e.*, the proportion of trials correctly identifying at least one effective intervention, assuming a risk ratio (RR) of 1.5 in at least one of the two comparisons (with participants allocated in a 2:2:1 ratio). In this context, the probability of falsely declaring at least one intervention effective when it is not (*i.e.*, RR = 1) would remain below 5% (false positives). All simulations will be conducted using R version 3.4.1. To address the potential for site-specific confounding, we will include the study site as a stratification factor in all primary analyses, using a Bayesian hierarchical modeling approach to account for between-site variation, which will allow for partial pooling and direct estimation of potential site-treatment interactions.

#### 2.11.2 Dissemination of Results

The dissemination committee will be formed with representatives from both units. The committee will be chaired by the principal investigator from unit 1, who will be responsible for the final decisions regarding the communication of the trial results to participants, healthcare professionals, the public, and other relevant groups (*e.g.*, through publications, reporting in databases, or other sharing data agreements), indicating any possible restrictions on publication. The guidelines for authorship will follow the principles of the 2019 Recommendations for the Conduct, Reporting, Editing, and Publication of Scholarly Work in Medical Journals drawn up by the ICMJE [56].

#### 2.11.3 Registration

The trial is registered at ClinicalTrials.gov with ID NCT06589544.

## 3 RESULTS AND DISCUSSION

Many people with cancer experience psychological issues, like depression. This could make it more difficult to cope with the burden of their illness, lower treatment acceptance, increase hospital stays, lower quality of life, and increase the risk of suicide [1, 5]. The detection of depression in cancer patients is challenging. Depression can easily be overlooked because symptoms of cancer and its treatment resemble neurovegetative symptoms of depression, such as fatigue, loss of appetite, and sleep disturbance.

Evaluating the feasibility and the cost-effectiveness of nonpharmacological interventions for cancer patients' quality of life and depressive symptoms, cognitive function, and other depression-related conditions is one of the main goals of this study. In this RCT, we will specifically compare standard TAU with rTMS and VR-COG *versus* TAU alone. The assessment of the cognitive functioning, depression-related conditions, and cost-effectiveness of the interventions under consideration will be another crucial aspect. The results of this study will allow us to obtain knowledge that can be translated into clinical practice regarding the feasibility and cost-effectiveness of an innovative therapeutic approach aimed at the treatment of depression in individuals suffering from oncological pathologies. This will have practical implications for the clinical health systems, considering the high costs linked to oncological diseases and depressive disorders. Despite this, the financial costs associated with depression are also substantial in patients with cancer. Cancer patients with a diagnosis of depression have annual healthcare costs more than double those of non-depressed patients, with higher charges occurring in major healthcare categories of ambulatory care, emergency department charges, and inpatient hospital settings [3].

The findings of the present RCT will be able to guide further study and clinical decisions in terms of both choice of treatment and the personalization of the same. The study will ultimately favour the application of safe and non-invasive interventions for the population with oncological pathologies and depressive disorders. The monitoring of the cost-effectiveness of the program, including both the screening and intervention phases, will allow policymakers to be guided in the implementation of this evidence in routine clinical practice.

### 3.1 Risk and Benefits

The use of immersive virtual reality (VR) may be associated with mild and transient side effects, including dizziness, nausea, headaches, visual fatigue, impaired limb coordination, diminished postural stability, a decreased sense of presence, and potentially inappropriate reactions to real-world environments. The side effects of rTMS are also mild and transient, and include headache, discomfort at the scalp stimulation site, tingling sensations or muscle twitching, and light-headedness. Nonetheless, significant adverse effects are not expected, as rTMS and VR are considered safe and well-tolerated procedures, with an extremely low risk of side effects [16, 57-60].

## 4 STUDY LIMITATIONS

This study is strengthened by its methodologically sound feasibility randomized controlled design, the adoption of standardized virtual reality-based cognitive remediation and rTMS protocols, a comprehensive multimodal evaluation encompassing several psychosocial outcomes, and consistency with established frameworks for the development and testing of complex interventions.

Potential limitations include challenges in participant recruitment within an oncological population presenting with mild to moderate depressive symptoms. These challenges may stem from clinical instability related to ongoing treatments (*e.g*., chemotherapy-related fatigue, pain, or cognitive burden), fluctuating symptom severity, competing medical appointments, and reduced motivation or engagement associated with depressive symptomatology. Additionally, concerns about time commitment, physical discomfort, or unfamiliarity with technology-based interventions may further limit willingness to participate. Given its pilot nature, the study is not intended to demonstrate definitive clinical efficacy, but rather to provide exploratory evidence and guide the design of subsequent, adequately powered trials.

## CONCLUSION

Many individuals with cancer experience depression, which often remains underdiagnosed and undertreated. It is linked to cognitive impairments, sleep disturbances, and functional disability, all contributing to poor clinical outcomes, worse prognosis, and increased healthcare costs. Although antidepressants are frequently used, their efficacy remains uncertain, and no specific guidelines exist for people with cancer.

The results of this study will provide clinically translatable knowledge regarding the feasibility and cost-effectiveness of an innovative therapeutic approach aimed at treating depression in individuals with oncological conditions. This will have practical implications for preventive and rehabilitative practice within healthcare systems, considering the high costs associated with cancer and depressive disorders.

The findings of the RCT may inform clinical decision-making, both in terms of treatment selection and personalization. The study will promote the implementation of a safe and novel non-pharmacological therapeutic approach using transcranial magnetic stimulation (rTMS) and a virtual reality-based cognitive remediation program to treat depression in cancer patients. The cost-effectiveness evaluation of the program, encompassing both the screening and intervention phases, will support policymakers in translating this evidence into routine clinical practice, and this will have practical implications for clinical health care systems.

## AUTHORS’ CONTRIBUTIONS

It is hereby acknowledged that all authors have accepted responsibility for the manuscript's content and consented to its submission. They have meticulously reviewed all results and unanimously approved the final version of the manuscript.

## Data Availability

The data of current study are available from corresponding author [G.K], on a reasonable request.

## LIST OF ABBREVIATIONS

rTMS: Repetitive transcranial magnetic stimulation
VR-COG: Virtual reality-based cognitive behavioural intervention
TAU: Treatment as usual
QoL: Quality of life
RCT: Randomised controlled trial
HAM-D-17: 17-item Hamilton Rating Scale for Depression
DS: Demoralization Scale
PTED: Post-traumatic Embitterment Disorder Self-rating Scale
BSI: Brief Symptom Inventory
ISI: Insomnia Severity Index
BRIAN: Biological Rhythms Interview for Assessment in Neuropsychiatry
ADL: Activities of Daily Living
PAIS: Psychosocial Adjustment to Illness
TAS-20: Toronto Alexithymia Scale 20-item
SCIP: Screen for Cognitive Impairment in Psychiatry
FAB: Frontal Assessment Battery
rMT: Resting motor threshold
AIOM-SIPO: Italian Association of Medical Oncology
GCP: Good Clinical Practice
IT: Information technology
EDC: Electronic data capture
SHELF: Sheffield Elicitation Framework
RR: Relative risk
LOCF: Last Observation Carried Forward protocol

## STANDARDS OF REPORTING

Consort guidelines will be followed.
